# Self-reported pain is associated with a poorer long-term prognosis in patients with myocardial infarction: A SWEDEHEART study

**DOI:** 10.1016/j.ijcha.2025.101719

**Published:** 2025-06-18

**Authors:** Lars Berglund, Ann-Sofie Rönnegård, Bertil Lindahl, Björn Äng., Torsten Gordh, Kristina Hambraeus, Johan Ärnlöv

**Affiliations:** a School of Health and Welfare, Dalarna University, Falun, Sweden; b Department of Public Health and Caring Sciences, Geriatrics, Uppsala University, Uppsala, Sweden; c Epistat AB, Uppsala, Sweden; d Center for Clinical Research Dalarna, Uppsala University, Sweden; e Department of Medical Sciences, Uppsala University, Uppsala, Sweden; f Department of Neurobiology, Care Sciences and Society, Division of Physiotherapy, Karolinska Institute, Huddinge, Sweden; g Department of Physical Education and Sport Science, Biomechanical and Ergonomic Laboratory, University of Thessaly, Trikala, Greece; h Department of Women’s and Children’s Health, Physiotherapy and Behavioural Medicine, Uppsala University, Sweden; i Department of Surgical Sciences, Pain Research, Uppsala University, Uppsala, Sweden; j Department of Cardiology, Falun Hospital, Falun, Sweden; k Department of Neurobiology, Care Sciences and Society, Division of Family Medicine and Primary Care, Karolinska Institute, Stockholm, Sweden

**Keywords:** Myocardial infarction, Pain, Prognosis

## Abstract

**Background:**

Pain is associated with cardiovascular risk, but its prognosis after myocardial infarction (MI) is less studied. We evaluated general pain post-MI as a marker for MACE (all-cause mortality, recurrent MI, or stroke) and all-cause mortality.

**Methods and results:**

We collected data from 98,441 MI patients (22–79 years) from the Swedish quality register SWEDEHEART. Pain, measured by EuroQol-5, was recorded one-year post-MI. Moderate pain was reported by 38.0 % of the patients and extreme pain by 5.0 %. During follow-up (up to 16.0 years (median 5.5 years)) there were 14,944 deaths and 24,910 MACEs.

In adjusted Cox regression models, moderate and extreme pain were associated with all-cause mortality in men (hazard ratio (HR) 1.24, 95 % confidence interval (CI) 1.19–1.29 and HR 1.70, 95 % CI 1.54–1.86, respectively,) and in women (HR 1.15, 95 % CI 1.08–1.23 and HR 1.31, 95 % CI 1.16–1.48, respectively,). The population attributable fraction (PAF) for moderate and extreme pain combined, with outcome all-cause mortality, was 8.3% for men and 6.3 % for women, similar to PAF for smoking, diabetes, and hypertension. Compared to all-cause mortality, HRs for MACE were somewhat lower in men and similar in women. For patients with lower cardiovascular risk defined, among other factors, by absence of chest pain, HRs were comparable to those in the main sample.

**Conclusions:**

Self-reported pain after MI was common and linked to increased cardiovascular risk, similar to that of smoking, diabetes, and hypertension. Clinicians may consider general pain in prognosis and treatment, even for patients without chest pain.

## Graphical abstract

**Figure:**
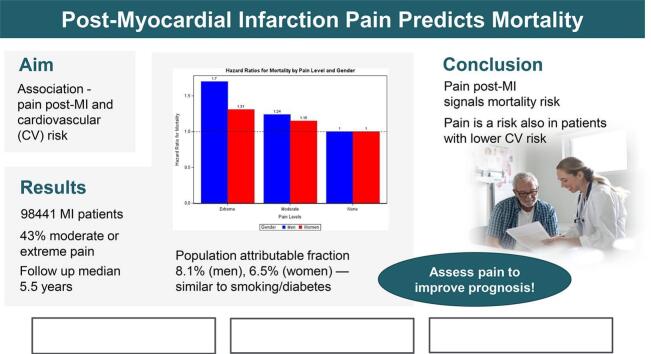

## 1 Background

Cardiovascular diseases such as myocardial infarction (MI) remains a leading cause of death worldwide. Moreover, improved treatment and prognosis after a first MI has led to an increasing prevalence of long-term MI survivors [[1], [2], [3]]. Despite this improvement, MI survivors still face a substantial long-term risk of recurrent MI, as well as other cardiovascular events and mortality [3,4].

Chronic pain, typically defined as pain persisting for more than 3 months, affects millions of individuals worldwide, contributing significantly to disability and reduced quality of life [5,6]. Although it has been linked to various health conditions, including cardiovascular diseases [7,8] and mortality [[8], [9], [10], [11]], its long-term effects on morbidity and mortality remain underexplored. Recently, there has been a paradigm change in how pain is understood and in the upcoming ICD 11 [6], chronic pain is defined as either “chronic primary pain - a disease in its own right”, or “chronic secondary pain - pain as a symptom of an underlying injury or illness”. This highlights the need for research into the broader impacts of chronic pain in relation to other health conditions.

Individuals with chronic pain and cardiovascular diseases share several common risk factors, including smoking, obesity [8], low physical activity levels [12], mental health issues, sleep disturbances, poor diet [13], and various socioeconomic factors [14] and emerging evidence suggest that chronic pain is a clinically important risk factor for incident atherosclerotic diseases such as MI [15]. The link between chronic pain and cardiovascular risk is likely bi-directional, with inflammation as one common factor. Both conditions show increased pro-inflammatory cytokines in blood, a key component of arteriosclerosis [16]. Chronic pain patients also exhibit elevated inflammatory markers in blood and cerebrospinal fluid, even in pain conditions traditionally considered as non-inflammatory pain conditions [17,18]. Inflammation may heighten peripheral nociceptor sensitivity [19,20] and activate central pain processing networks, worsening pain perception [21]. Chronic psychological stress, common in both conditions [22,23], can trigger inflammatory responses [24], contributing to systemic inflammation and increasing cardiovascular and overall disease risk [24,25]. Pain itself acts as a stressor, alongside other life stressors, amplifying this effect [23,24].

Little is known regarding the prognostic importance of pain after an MI. Understanding the role of pain in post-MI outcomes could lead to more comprehensive treatment approaches, where pain management is integrated into secondary prevention strategies which could improve patient prognosis and quality of life.

To address this research gap we have previously conducted a study encompassing 18,376 patients from a Swedish national registry of MI patients [26] where we reported higher mortality rates in patients experiencing moderate and extreme general pain one-year post-MI. The design and the aims of the present study represent an extension and improvement of this previous study by several key factors: 1. The present study is based on a more than five-fold larger sample size and almost 15 times as many deaths during follow-up, compared to our previous study, for evaluation of the association between general pain and outcomes. The larger study sample allows us to study effect modifications (interactions) analyses between pain and demographic factors such as sex or age, and to estimate associations between pain and outcomes in individuals with lower estimated cardiovascular risk defined, among other factors, by absence of chest pain. 2. The present study has an almost double follow-up time with up to 16 years follow-up period allowing for better estimates of long-term prognosis associated with pain. 3. The present study also includes data on both fatal and non-fatal major adverse cardiovascular events (MACE; the composite of all-cause mortality, recurrent MI, or stroke) in addition to the mortality data presented in the previous study adding to the clinical relevance of the results. A further aim of the present study was to describe how pain categories were associated with known risk factors of mortality after an MI.

## 2 Methods

### 2.1 Data availability

The authors are not authorized to share data from Swedish quality registers.

### 2.2 Study sample

This prospective cohort study included Swedish patients aged 22–79 years who experienced an MI event (International Classification of Diseases, Tenth Revision [ICD-10] code I21) between December 1, 2004, and December 31, 2020. If multiple MI events occurred for a patient during this period, only the first was included. Data were obtained from the Swedish quality register, SWEDEHEART (Swedish Web System for Enhancement and Development of Evidence-Based Care in Heart Disease Evaluated According to Recommended Therapies) and from the SWEDEHEART-sub register for cardiac rehabilitation (CR), SWEDEHEART-CR, which collects data at out-patient visits to CR-nurse or physician at 2 months (timeframe 6–10 weeks) and 1 year (timeframe 11–13 months) after discharge.

The study was approved by the Ethical Review Board in Stockholm (approval numbers 2012-/60–31/2 and 2020–04252).

### 2.3 Study exposure

Data on pain categories were collected from the 1-year follow-up questionnaire registered in SWEDEHEART-CR. The questionnaire includes the EuroQol-5 dimension (EQ-5D) instrument with five self-reported dimensions of health (one of them pain) [27]. The pain dimension entails three response options; “I have no pain or discomfort”, "I have moderate pain or discomfort” or “I have extreme pain or discomfort”. In addition, data on EQ-5D pain measurement from the follow-up visit 2 months post-MI were used to follow pain over time.

### 2.4 Covariates

Data on patient characteristics at hospital discharge were: year of hospital discharge, age, sex, Body Mass Index (BMI), diabetes, hypertension, hyperlipidemia, creatinine levels, previous percutaneous coronary intervention (PCI), PCI during hospital stay, coronary artery bypass grafting (CABG) during hospital stay, previous MI, previous stroke, previous congestive heart failure, and type of MI (non-ST segment elevation MI (NSTEMI) or a ST segment elevation MI (STEMI)). Data on the prevalence of diabetes, hypertension, hyperlipidemia, previous MI, and previous stroke are entered into the registry based on patients' reported histories and, when possible, verified through medical record audits. While there is a risk of incorrect reporting, regular monitoring of the registry data ensures its accuracy, with consistent confirmation of over 95 % agreement with medical records [28,29]. Data on BMI were based on measured weight and height, with the subject standing, to the nearest 0.1 kg and 1 cm, respectively. If it was not possible to conduct measurements, self-reported data were registered if the patient was able to provide them. If not, the data was registered as missing.

Chest pain and smoking were recorded at the 1-year visit. Chest pain was classified as no pain/CCS class I/CCS class II/ CCS class III/ CCS class IV/non ischemic chest pain according to the Canadian Cardiovascular Society Angina Grade CCS. Smoking was categorized as Never/Previous (stopped smoking > 1 month ago) or Current smoker.

### 2.5 Outcomes

Mortality and morbidity data were collected from the nationwide cause-of-death register and patient register, respectively, held by the National Board of Health and Welfare in Sweden. The patient register includes all discharge diagnoses (ICD-10 codes) in all patients hospitalized in Sweden and has excellent completeness and accuracy for cardiovascular diagnoses [30].

Included outcomes were all-cause mortality and major adverse cardiovascular events (MACE; the composite of all-cause mortality, recurrent MI, or stroke). The study index date was the date for the 1-year visit post-MI, follow-up for each outcome was until an event occurred or January 16th, 2022, whichever occurred first, and time to event was the difference between these two dates.

### 2.6 Statistical analysis

Continuous variables were presented as means with standard deviations, while categorical variables were represented as counts and percentages, in total and by pain categories stratified by sex. Comparisons of pain groups were made using the Kruskal-Wallis test for continuous variables and a chi-squared test for categorical variables.

#### 2.6.1 Missing data and multiple imputation

Missing data ranged from 0 % to 8 %, with the highest percentages for BMI (7 %) and diabetes (8 %). A total of 80 720 patients (82.0 %) had complete data across all analysis variables.

To address missing data, we applied multiple imputation using chained equations (MICE) under the assumption of Missing at Random (MAR)[31]. We implemented this procedure using the SAS procedure MI with the FCS (Fully Conditional Specification) method, which allows flexible modeling of different variable types (continuous and categorical) in the imputation process.

The imputation model included all covariates used in the statistical analyses. Dichotomous variables (sex, diabetes, hypertension, hyperlipidemia, previous PCI, PCI during hospital stay, CABG during hospital stay, previous MI, previous stroke, previous congestive heart failure, and type of MI) were modeled using logistic regression, while categorical variables with more than two levels (smoking and chest pain) were imputed using multinomial regression, and continuous variables (age, BMI and creatinine levels) were modeled using linear regression. The missing values for each variable were imputed conditionally on other observed variables using a chained equations approach. The algorithm iteratively imputes each variable one at a time, conditioning on the others, until convergence is reached. We used a default burn-in period of 20 iterations, which is recommended to ensure that the imputation process has sufficiently converged to stable estimates, before collecting the final imputations.

The number of imputed datasets was the percentage of incomplete cases rounded up to an integer (=19). After imputation, we pooled the results across the imputed datasets using Rubin’s rules to account for the variability within and between the imputed datasets. This method ensures that the final parameter estimates account for both the uncertainty of the imputed values and the variability of the observed data.

#### 2.6.2 Pain in relation to outcomes

Due to significant interactions between pain and sex on outcome all-cause mortality all analyses were stratified by sex. We used Cox regression models to estimate associations between pain and risk of all-cause mortality and MACE. Results were presented as hazard ratios (HRs) with 95 % confidence intervals (CIs) for moderate pain and for extreme pain with no pain as reference category (pain was registered 1-year post-MI). In a basic model, the associations were adjusted for age, and in an extended model adjustments were made for the following variables: age, year of hospital stay, diabetes, hypertension, hyperlipidemia, creatinine level, previous PCI, PCI during hospital stay, previous MI, previous stroke, previous congestive heart failure, diagnosis at discharge (NSTEMI/STEMI), CABG, BMI, chest pain (1 year post-MI) and smoking (1 year post-MI). All covariates were registered at hospital discharge if not otherwise indicated. For age, BMI, and creatinine levels we employed 3-knotted restricted cubic splines. For all-cause mortality we estimated the population attributable fraction (PAF) associated with pain (moderate pain and extreme pain combined), current smoking, diabetes, and hypertension. The PAF was estimated as p*(1–1/HR) where p was the proportion of exposed patients and HR was the adjusted hazard ratio for the exposure from the extended model.

We sought to explore the associations between pain and outcomes in patients with estimated lower cardiovascular risk than the general population of MI patients. Thus, analyses were restricted to patients under 68 years of age who had no chest pain according to the Canadian Cardiovascular Society Angina Grade (CCS) 1 year post-MI, normal creatinine levels (<100 µmol/L for men and < 90 µmol/L for women), were not current smokers, did not have diabetes, had a BMI between 23 kg/m^2^ and 33 kg/m^2^ and did not have previous PCI, MI or CHF. The Cox regression model for this analysis, stratified by sex, was adjusted for age, year of hospital stay, smoking status (never or previous smoker one year after hospital stay), hypertension, hyperlipidemia, creatinine level, PCI during hospital stay, diagnosis at discharge (NSTEMI/STEMI), CABG and BMI (extended model 2).

The assumptions of proportional hazards (PH) were examined with plots of log[−log(survival)] versus log of survival time. Due to violations of PH assumption for pain, we estimated weighted averages of the hazard ratios over the entire follow-up period [32]. To that end we applied inverse probability exposure weighting (IPW). In this approach, each patient was weighed in Cox regression models by the inverse of the probability of being in the pain category that he/she in fact was. The probability was estimated from a multinomial logistic regression model with pain categories as dependent variable and the covariates in the basic model, the extended model, or the extended model 2, respectively, as predictor variables. Estimation of standard errors for hazard ratios was made with a sandwich estimator.

#### 2.6.3 Sensitivity analyses

To explore the putative effects of reverse causation in the basic model and in the extended model, we conducted sensitivity analyses for all-cause mortality and MACE where patients with a shorter follow-up time than 2 years after the 1-year visit post-MI were excluded.

In another sensitivity analysis, we analyzed the associations between pain and all-cause mortality in the extended model by sex for patients with complete data on all covariates (n = 80 720).

We calculated the E-value to assess the potential impact of unmeasured confounders on the observed association between pain and all-cause mortality [33]. The E-value quantifies the strength of unmeasured confounders needed to explain away the observed associations.

#### 2.6.4 Interaction analyses

Interactions between pain and age, and between pain and sex, were examined in Cox proportional hazards regression models for all-cause mortality and for MACE, including the covariates from the extended model, while accounting for multiple imputation [34].

#### 2.6.5 Visualization

We visualized survival probabilities for all-cause mortality and for MACE using Kaplan-Meier survival curves by pain categories stratified by sex. Comparisons of Kaplan-Meier survival curves between pain categories were made with the log-rank test.

For all-cause mortality and MACE we estimated hazard ratios of pain categories stratified by sex, with adjustment according to the extended model, over time (HRt) as antilog of (time-fixed Cox regression coefficients under PH + scaled Schoenfeld’s residuals) and fitted locally estimated scatterplot smoothing (LOESS) curves for HRt versus time. These time-varying hazard ratios were graphically depicted over the follow-up period to illustrate the temporal effect of pain on outcomes.

#### 2.6.6 Significance level and software

Statistical tests and confidence intervals were two-sided (where applicable). Results with p < 0.05 were considered statistically significant without adjustments for multiplicity. All statistical analyses were performed using SAS version 9.4 (SAS Institute, Cary, NC, USA).

## 3 Results

### 3.1 Patient characteristics

**Fig. 1:**
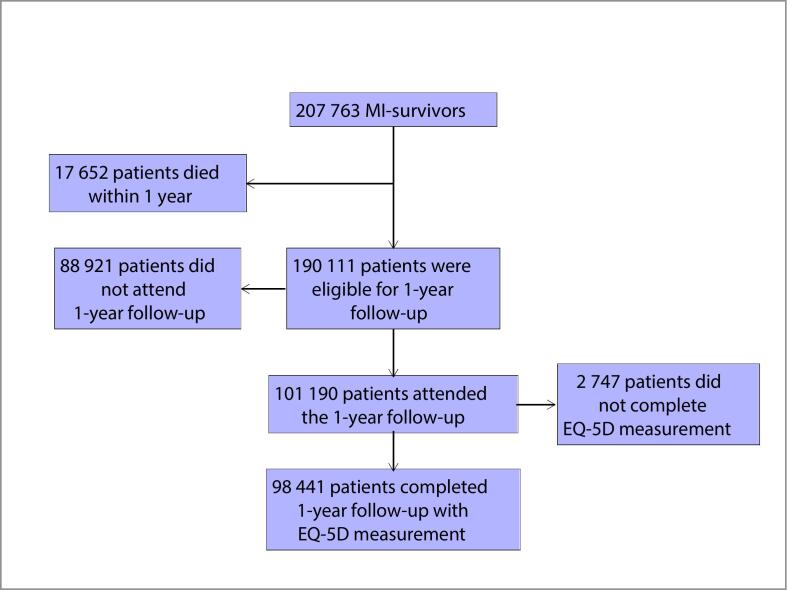
Flowchart of patient selection.

During the study period 207,763 MI-survivors were registered in the SWEDEHEART registry and 190,111 were alive 1-year post-MI (Fig. 1). We included 98,441 patients who attended the 1-year post-MI visit and performed an EQ-5D measurement of pain. Included and nonincluded patients who were alive 1-year post-MI differed for age (mean ages 63.3 and 67.2 years, respectively), sex (25.4 % and 32.2 % women, respectively), and incidence rates of all-cause mortality from one-year after hospital discharge (248.8 and 593.5 per 10,000 person-years, respectively).

Table 1 (for men) and Table 2 (for women) show significant differences in clinical characteristics, comorbidities, and procedural history across patients with different pain levels. Patients with moderate or extreme pain tended to have higher rates of comorbid conditions (e.g., diabetes, hypertension) and higher BMI compared to those with no pain. Additionally, there were more smokers and more patients with chest pain in the moderate and extreme pain categories compared with patients with no pain. Furthermore, Table 1, Table 2 show that men and women differ in the proportions of pain categories. The proportions of patients with moderate and extreme pain were 35.6 % and 4.2 %, respectively, for men while for women the proportions were 46.1 % and 7.7 %, respectively (p < 0.0001). Among the 51,337 patients who reported no pain at the 2-month follow-up post-MI, 73 % remained pain-free at 1-year post-MI. Similarly, 65 % of those who experienced pain at 2 months post-MI continued to report pain at 1-year follow-up. Among those with new pain 1-year post-MI, 80 % did not exhibit chest pain (based on CCS), while 94 % of patients who remained pain-free did not report chest pain.

**Table 1:** Characteristics of the study sample for men at hospital discharge in total and by EQ-5D pain categories 1  year after MI.

	Pain categories 1-year visit				
	No pain (n = 44245, 60.2 %)	Moderate pain (n = 26149, 35.6 %)	Extreme pain (n = 3056, 4.2 %)	Total (n = 73450)	P-value
Year of hospital discharge					0<.0001§
2004–2009	8880 (20.1)	5681 (21.7)	605 (19.8)	15,166 (20.6)	
2010–2015	17,913 (40.5)	9796 (37.5)	1186 (38.8)	28,895 (39.3)	
2016–2020	17,452 (39.4)	10,672 (40.8)	1265 (41.4)	29,389 (40.0)	
Age, y, mean (SD)	62.6 (9.0)	63.4 (8.8)	62.4 (9.0)	62.9 (8.91)	0<.0001†
BMI, kg/m^2^, mean (SD)	27.5 (4.0)	28.2 (4.4)	28.9 (5.0)	27.8 (4.18)	0<.0001†
Diabetes	6233 (15.2)	5415 (22.5)	811 (28.8)	12,459 (18.4)	0<.0001§
Hypertension	18,757 (42.6)	13,243 (50.9)	1635 (53.8)	33,635 (46.0)	0<.0001§
Hyperlipidaemia	43,229 (98.5)	25,412 (98.1)	2943 (97.5)	71,584 (98.3)	0<.0001§
Creatinine, µmol/L, mean (SD)	87.4 (39.1)	90.1 (49.2)	91.5 (55.0)	88.5 (43.73)	0.1638†
Previous MI	6977 (15.8)	5826 (22.4)	855 (28.2)	13,658 (18.7)	0<.0001§
Previous stroke	1429 (3.2)	1352 (5.2)	193 (6.4)	2974 (4.1)	0<.0001§
Previous CHF	2346 (5.3)	2018 (7.7)	331 (10.8)	4695 (6.4)	0<.0001§
Previous PCI	5815 (13.2)	4969 (19.1)	719 (23.7)	11,503 (15.7)	0<.0001§
CABG	2616 (5.9)	1531 (5.9)	172 (5.6)	4319 (5.9)	0.7928§
Diagnosis					0<.0001§
NSTEMI	25,767 (58.3)	16,303 (62.4)	1957 (64.1)	44,027 (60.0)	
STEMI	18,445 (41.7)	9829 (37.6)	1098 (35.9)	29,372 (40.0)	
PCI	37,527 (84.8)	21,516 (82.3)	2467 (80.7)	61,510 (83.7)	0<.0001§
Smoking*					0<.0001§
Current	4690 (10.6)	3550 (13.6)	627 (20.6)	8867 (12.1)	
Never	16,263 (36.8)	7692 (29.5)	697 (22.8)	24,652 (33.6)	
Previous (stopped > 1 mo ago)	23,232 (52.6)	14,871 (56.9)	1727 (56.6)	39,830 (54.3)	
Chest pain*					0<.0001§
CCS I	1871 (4.2)	3105 (11.9)	336 (11.0)	5312 (7.2)	
CCS II	360 (0.8)	1269 (4.9)	193 (6.3)	1822 (2.5)	
CCS III	66 (0.1)	367 (1.4)	122 (4.0)	555 (0.8)	
CCS IV	16 (0.0)	74 (0.3)	36 (1.2)	126 (0.2)	
No chest pain	41,158 (93.0)	20,006 (76.5)	2185 (71.5)	63,349 (86.3)	
Nonischemic chest pain	767 (1.7)	1324 (5.1)	184 (6.0)	2275 (3.1)	

Data are given as number (percentage) unless otherwise indicated. EQ-5D denotes EuroQol-5 dimension instrument; MI, myocardial infarction; BMI, body mass index; CHF, congestive heart failure; CABG, coronary artery bypass grafting; NSTEMI, non-ST segment elevation MI; STEMI, ST-segment–elevation MI; PCI, percutaneous coronary intervention and CCS, Canadian Cardiovascular Society.

* Measured at the visit 1 year post-MI.

§ Chi-Square p-value.

† Kruskal-Wallis p-value.

**Table 2:** Characteristics of the study sample for women at hospital discharge in total and by EQ-5D pain categories 1  year after MI.

	Pain categories 1-year visit				
	No pain (n = 11537, 46.2 %)	Moderate pain (n = 11533, 46.1 %)	Extreme pain (n = 1921, 7.7 %)	Total (n = 24991)	P-value
Year of hospital discharge					0.0008§
2004–2009	2360 (20.5)	2597 (22.5)	420 (21.9)	5377 (21.5)	
2010–2015	4631 (40.1)	4371 (37.9)	746 (38.8)	9748 (39.0)	
2016–2020	4546 (39.4)	4565 (39.6)	755 (39.3)	9866 (39.5)	
Age, y, mean (SD)	64.2 (8.9)	65.2 (8.4)	64.9 (8.6)	64.7 (8.65)	0<.0001†
BMI, kg/m^2^, mean (SD)	26.9 (4.9)	28.1 (5.5)	29.4 (6.0)	27.7 (5.31)	0<.0001†
Diabetes	1610 (15.1)	2507 (23.5)	547 (30.6)	4664 (20.2)	<.0001§
Hypertension	5499 (47.9)	6506 (56.7)	1121 (58.6)	13,126 (52.8)	<.0001§
Hyperlipidaemia	11,078 (97.4)	11,003 (96.9)	1810 (96.4)	23,891 (97.1)	0.0142§
Creatinine, µmol/L, mean (SD)	70.3 (33.3)	74.2 (42.4)	78.4 (55.3)	72.7 (39.71)	0<.0001†
Previous MI	1275 (11.1)	1876 (16.3)	402 (21.0)	3553 (14.3)	<.0001§
Previous stroke	411 (3.6)	536 (4.7)	122 (6.4)	1069 (4.3)	<.0001§
Previous CHF	471 (4.1)	737 (6.4)	171 (8.9)	1379 (5.5)	<.0001§
Previous PCI	992 (8.6)	1466 (12.7)	314 (16.4)	2772 (11.1)	<.0001§
CABG	457 (4.0)	473 (4.1)	62 (3.2)	992 (4.0)	0.1918§
Diagnosis					<.0001§
NSTEMI	7220 (62.6)	7563 (65.6)	1332 (69.4)	16,115 (64.5)	
STEMI	4309 (37.4)	3960 (34.4)	588 (30.6)	8857 (35.5)	
PCI	8565 (74.2)	8464 (73.4)	1394 (72.6)	18,423 (73.7)	0.1672§
Smoking*					<.0001§
Current	1572 (13.6)	1719 (14.9)	385 (20.1)	3676 (14.7)	
Never	4225 (36.7)	3830 (33.2)	525 (27.4)	8580 (34.4)	
Previous (stopped > 1 mo ago)	5727 (49.7)	5970 (51.8)	1006 (52.5)	12,703 (50.9)	
Chest pain*					<.0001§
CCS I	679 (5.9)	1558 (13.5)	263 (13.7)	2500 (10.0)	
CCS II	114 (1.0)	568 (4.9)	122 (6.4)	804 (3.2)	
CCS III	17 (0.1)	175 (1.5)	68 (3.5)	260 (1.0)	
CCS IV	5 (0.0)	25 (0.2)	19 (1.0)	49 (0.2)	
No chest pain	10,447 (90.6)	8576 (74.4)	1318 (68.6)	20,341 (81.4)	
Nonischemic chest pain	272 (2.4)	628 (5.4)	131 (6.8)	1031 (4.1)	

Data are given as number (percentage) unless otherwise indicated. EQ-5D denotes EuroQol-5 dimension instrument; MI, myocardial infarction; BMI, body mass index; CHF, congestive heart failure; CABG, coronary artery bypass grafting; NSTEMI, non-ST segment elevation MI; STEMI, ST-segment–elevation MI; PCI, percutaneous coronary intervention and CCS, Canadian Cardiovascular Society.

* Measured at the visit 1 year post-MI.

§ Chi-Square p-value.

† Kruskal-Wallis p-value.

Among the 88,921 patients who were alive at one year but did not attend the 1-year follow-up, 11,729 (13.2 %) patients completed an EQ-5D measurement at the two-month follow-up. Of these patients, 39.3 % reported experiencing moderate pain, while 5.1 % reported experiencing extreme pain. Among the 98,441 patients included at the one-year follow-up, 88,880 (90.3 %) completed an EQ-5D measurement at the two-month follow-up and of those 37.4 % reported moderate pain and 3.8 % reported extreme pain.

### 3.2 Pain and outcomes

The median (maximum) follow-up period for survival was 5.5 (16.0) years, and 14,944 deaths occurred in 600,760 person-years (incidence rate: 248.8 per 10,000 person-years). Of these, 6 918 events (incidence rate: 198.8 per 10,000 person-years) were in the no pain group, 6 837 events (incidence rate: 303.5 per 10,000 person-years) in the moderate pain group, 1 189 (incidence rate: 432.7 per 10,000 person-years) events in the extreme pain group. The median (maximum) follow-up period for major adverse cardiovascular events (MACE) was 5.5 (16.0) years and 24,910 MACEs occurred in 544,026 person-years. Of these, 12 103 events (incidence rate: 378.9 per 10,000 person-years) were in the no pain group, 11,040 events (incidence rate: 550.3 per 10,000 person-years) in the moderate pain group, 1 767 (incidence rate: 737.5 per 10,000 person-years) events in the extreme pain group.

**Fig. 2:**
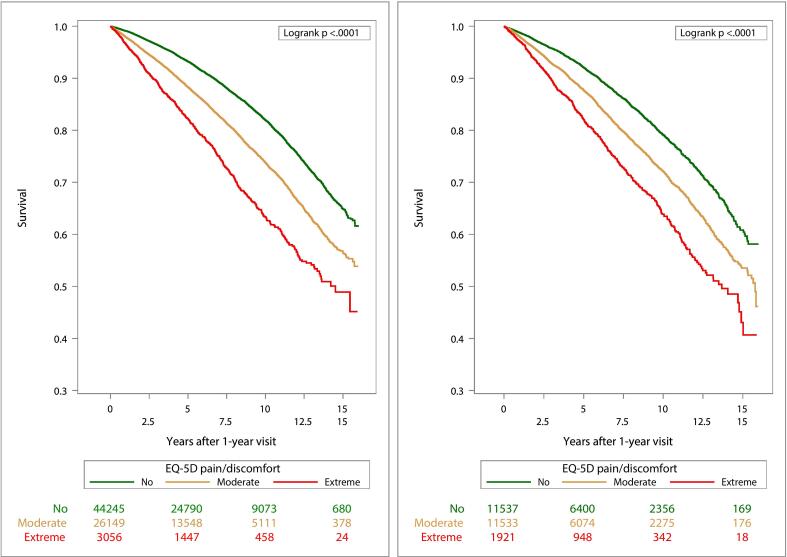
Kaplan-Meier estimates of all-cause mortality from 1 year after a myocardial infarction (MI) and number of subjects at risk by pain categories 1 year after MI for men (left) and women (right).

**Fig. 3:**
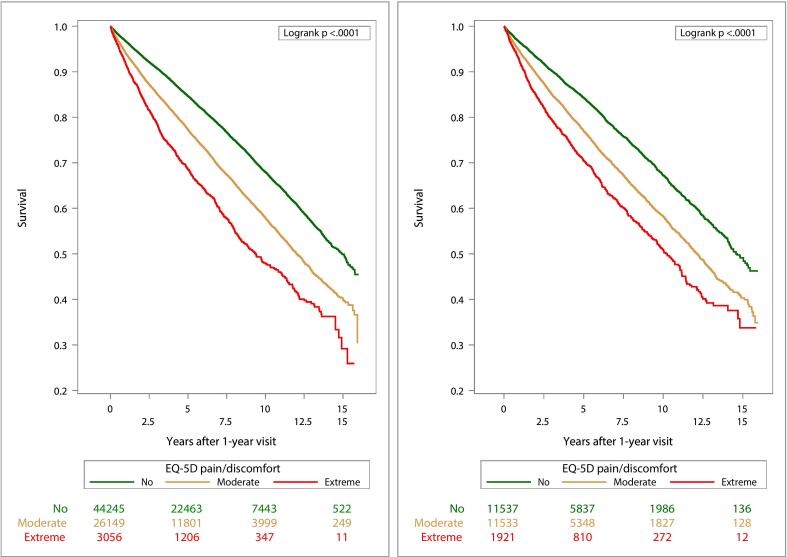
Kaplan-Meier estimates of MACE (the composite of all-cause mortality, non-fatal MI, or non-fatal stroke) from 1 year after a myocardial infarction (MI) and number of subjects at risk by pain categories 1 year after MI for men (left) and women (right).

**Fig. 4:**
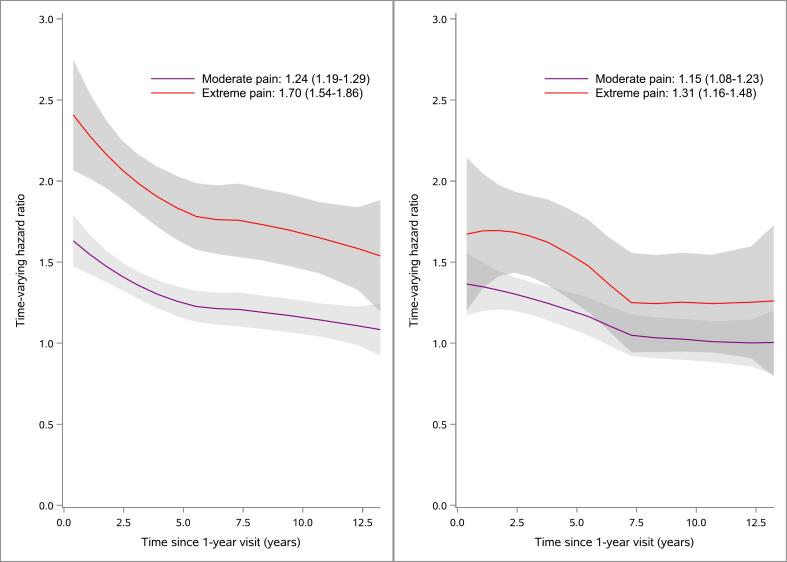
Time-varying hazard ratios (HR) for men (left) and women (right) illustrating the relative hazard for all-cause mortality for moderate pain and extreme pain compared with patients without pain, with 95 % confidence intervals (CI) and time-fixed overall HR assuming proportional hazards. HRs were adjusted for age, body mass index, creatinine, diabetes, hypertension, hyperlipidemia, previous MI, previous stroke, previous congestive heart failure, diagnosis (ST-segment elevation MI/non-ST-segment elevation MI), percutaneous coronary intervention, coronary artery bypass grafting, year of hospital discharge, chest pain (1-year visit), and smoking (1-year visit). All but the two last covariates were measured at hospital discharge.

**Fig. 5:**
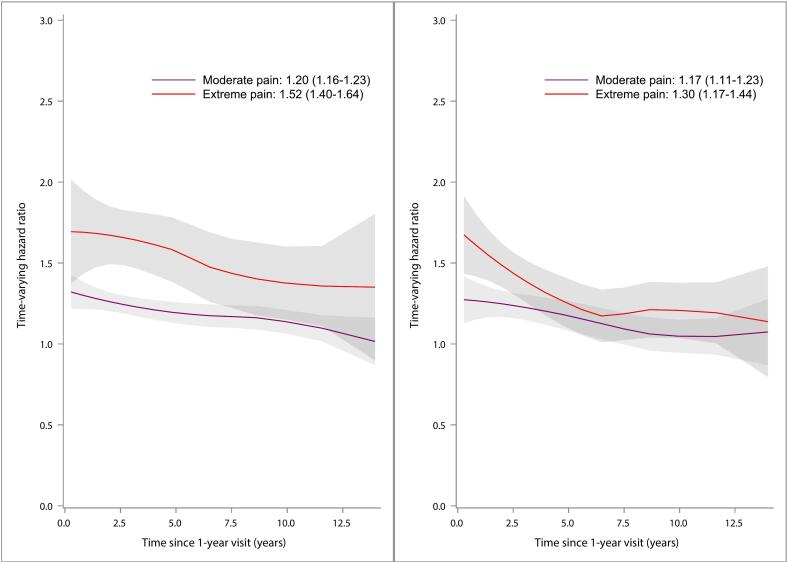
Time-varying hazard ratios (HR) for men (left) and women (right) illustrating the relative hazard for MACE (the composite of all-cause mortality, recurrent MI, or stroke) for moderate pain and extreme pain compared with patients without pain, with 95 % confidence intervals (CI) and time-fixed overall HR (assuming proportional hazards). HRs were adjusted for age, body mass index, creatinine, diabetes, hypertension, hyperlipidemia, previous MI, previous stroke, previous congestive heart failure, diagnosis (ST-segment elevation MI/non-ST-segment elevation MI), percutaneous coronary intervention, coronary artery bypass grafting, year of hospital discharge, chest pain (1-year visit), and smoking (1-year visit). All but the two last covariates were measured at hospital discharge.

Survival time and MACE-free time by pain categories are presented in Kaplan-Meier curves stratified by sex (Fig. 2, Fig. 3). Time-varying HRs for moderate and extreme pain categories, with no pain as the reference category, for outcomes all-cause mortality (Fig. 4) and MACE (Fig. 5) are presented by sex. Each HR was adjusted according to the extended model. Over the entire follow-up period, patients with moderate and extreme pain had higher rates of all-cause mortality than patients without pain. The excess rates declined over time and were most pronounced during the first five years after the visit at 1-year post-MI.

**Fig. 6:**
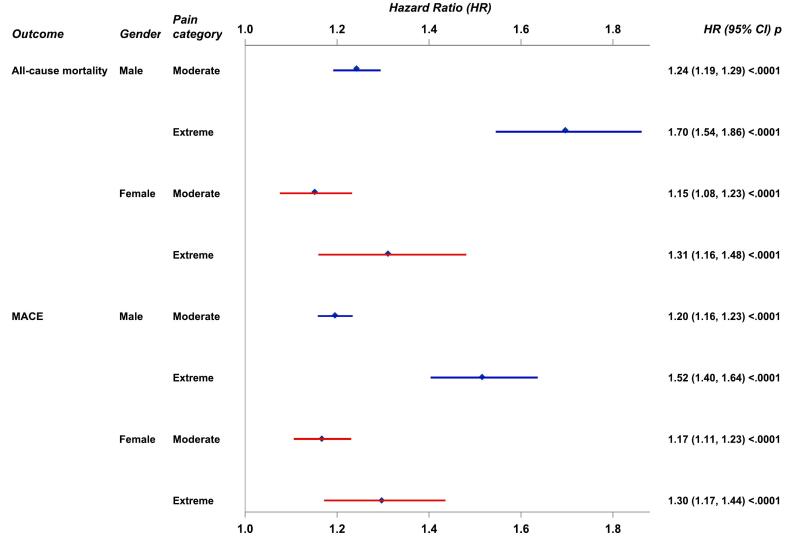
Hazard ratios (HR) with 95% confidence intervals (CI) by sex for pain categories moderate and extreme pain with no pain as reference category for outcomes all-cause mortality and MACE (the composite of all-cause mortality, recurrent MI, or stroke). HRs were adjusted for age, body mass index, creatinine, diabetes, hypertension, hyperlipidemia, previous MI, previous stroke, previous congestive heart failure, diagnosis (ST-segment elevation MI/non-ST-segment elevation MI), percutaneous coronary intervention, coronary artery bypass grafting, year of hospital discharge, chest pain (1-year visit) and smoking (1-year visit). All but the two last covariates were measured at hospital discharge.

The forest plot (Fig. 6) shows average HRs over the complete follow-up time for moderate and extreme pain categories, with no pain as the reference category, for outcomes all-cause mortality and MACE, stratified by sex. Each HR was adjusted according to the extended model and presented with a 95 % confidence interval (CI).

For all-cause mortality in male patients, the HR for moderate pain was 1.24 (95 % CI: 1.19, 1.29) compared to those with no pain, and for extreme pain the HR was 1.70 (95 % CI: 1.54, 1.86). In female patients, the HR for all-cause mortality was 1.15 (95 % CI: 1.08, 1.23) for moderate pain and 1.31 (95 % CI: 1.16, 1.48) for extreme pain. The association between extreme pain and all-cause mortality was more pronounced in males than in females.

For MACE in male patients, the HR for moderate pain was 1.20 (95 % CI: 1.16, 1.23) compared to those with no pain, and for extreme pain, the HR was 1.52 (95 % CI: 1.40, 1.64). In female patients, the HR for MACE was 1.17 (95 % CI: 1.11, 1.23) for moderate pain and 1.30 (95 % CI: 1.17, 1.44) for extreme pain.

Table 3 presents HR with 95 % CI for the basic model and for the extended model for outcomes all-cause mortality and MACE by sex. Further, Table 3 shows HRs by sex for the basic model and for the extended model for outcomes all-cause mortality and MACE for the sensitivity analysis where patients with a shorter follow-up time than 2 years after the 1-year visit were excluded. The sensitivity analysis resulted in somewhat lower HRs than in the main analysis for men (HR for all-cause mortality in the extended model was 1.70 (95 % CI: 1.54, 1.86) for extreme pain in the main analysis and 1.56 (95 % CI:1.40, 1.73) in the sensitivity analysis) but for women HRs were similar in the main analysis and the sensitivity analysis.

**Table 3:** Hazard ratios with 95 % confidence intervals (CI) by sex for pain categories moderate and extreme pain with no pain as reference category for outcomes all-cause mortality and MACE (the composite of all-cause mortality, recurrent myocardial infarction (MI), or stroke).

Sex	Sample	Model	Pain category	All-cause mortality hazard ratio (95 % CI)	MACE hazard ratio (95 % CI)
Male	Main sample	Basic model	Moderate pain	1.48 (1.43, 1.54)	1.42 (1.38, 1.47)
			Extreme pain	2.17 (2.01, 2.36)	1.94 (1.82, 2.07)
	Sensitivity sample	Basic model	Moderate pain	1.39 (1.33, 1.45)	1.33 (1.28, 1.38)
			Extreme pain	1.96 (1.78, 2.15)	1.75 (1.61, 1.90)
	Main sample	Extended model*	Moderate pain	1.24 (1.19, 1.29)	1.20 (1.16, 1.23)
			Extreme pain	1.70 (1.54, 1.86)	1.52 (1.40, 1.64)
	Sensitivity sample	Extended model	Moderate pain	1.18 (1.13, 1.24)	1.16 (1.12, 1.21)
			Extreme pain	1.56 (1.40, 1.73)	1.42 (1.29, 1.57)

Female	Main sample	Basic model	Moderate pain	1.37 (1.29, 1.46)	1.38 (1.31, 1.45)
			Extreme pain	1.85 (1.67, 2.06)	1.73 (1.59, 1.89)
	Sensitivity sample	Basic model	Moderate pain	1.33 (1.24, 1.43)	1.32 (1.24, 1.40)
			Extreme pain	1.75 (1.56, 1.97)	1.52 (1.36, 1.69)
	Main sample	Extended model*	Moderate pain	1.15 (1.08, 1.23)	1.17 (1.11, 1.23)
			Extreme pain	1.31 (1.16, 1.48)	1.30 (1.17, 1.44)
	Sensitivity sample	Extended model	Moderate pain	1.13 (1.05, 1.22)	1.14 (1.07, 1.21)
			Extreme pain	1.31 (1.14, 1.50)	1.20 (1.06, 1.36)

Base model: Adjustment for age.

Extended model: Adjustments for age, body mass index, creatinine, diabetes, hypertension, hyperlipidemia, previous MI, previous stroke, previous congestive heart failure, diagnosis (ST-segment elevation MI/non-ST-segment elevation MI), percutaneous coronary intervention,

coronary artery bypass grafting, year of hospital discharge, chest pain (one-year post-MI), and smoking (one-year post-MI). All but the two last covariates were measured at hospital discharge.

Sensitivity sample: Patients with shorter follow-up time than 2 years after the 1-year visit post-MI were excluded.

* This model is also presented in Fig. 6.

In the sensitivity analysis, where we used data from patients with complete information for all covariates, the HR for all-cause mortality in the extended model for men (n = 60,437) was 1.70 (95 % CI: 1.54, 1.89) for extreme pain and 1.24 (95 % CI: 1.19, 1.30) for moderate pain. For women (n = 20,283), the corresponding HRs were 1.33 (95 % CI: 1.16, 1.52) for extreme pain and 1.15 (95 % CI: 1.07, 1.24) for moderate pain. Thus, HRs in this sensitivity analysis were almost identical to HRs in the main analysis.

The calculated E-values for extreme pain of 2.79 and 1.70 for men and women, respectively, for outcome all-cause mortality in the extended model suggest stability of findings. Furthermore, these E-values exceed all hazard ratios (except the hazard ratio for current smoking in women) for the measured putative confounders, indicating that unmeasured confounders are unlikely to substantially influence the results.

PAF for pain (moderate pain and extreme pain combined) and outcome all-cause mortality was 8.3 % and 6.3 % for men and women, respectively. The corresponding PAF for current smoking was 6.0 % in men and 7.5 % in women, for diabetes 7.4 % and 9.3 %, and for hypertension 5.3 % and 5.1 %, respectively.

In the analysis, where we explored associations between pain categories and outcomes in patients with lower cardiovascular risk defined, among other factors, by absence of chest pain, (19, 145 men and 4 129 women), adjusted HRs for all-cause mortality and for MACE (Table 4) were similar to the adjusted HRs in the main sample.

**Table 4:** Hazard ratios with 95% confidence intervals (CI) by sex for pain categories moderate and extreme pain with no pain as reference category for outcomes all-cause mortality and MACE (the composite of all-cause mortality, recurrent myocardial infarction (MI), or stroke) for patients with lower cardiovascular risk.*

Sex	n	Pain category	All-cause mortality hazard ratio† (95 % CI)	MACE hazard ratio† (95 % CI)
Male	19,145	Moderate pain	1.20 (1.05, 1.36)	1.14 (1.04, 1.24)
		Extreme pain	1.64 (1.17, 2.31)	1.67 (1.33, 2.09)

Female	4129	Moderate pain	1.27 (0.97, 1.65)	1.22 (1.02, 1.45)
		Extreme pain	1.38 (0.80, 2.37)	1.49 (1.03, 1.45)

* Analyses were restricted to patients under 68 years of age who had normal creatinine values (<100 µmol/L for men and <90 µmol/L for women), no chest pain according to the Canadian Cardiovascular Society Angina Grade 1-year post-MI, were not current smokers, did not have diabetes, had a body mass index between 23 kg/m^2^ and 33 kg/m^2^ and did not have previous percutaneous coronary intervention, MI or congestive heart failure.

† Hazard ratios were adjusted for age, year of hospital stay, smoking status (never or previous smoker 1-year post-MI), hypertension, hyperlipidemia, creatinine level, percutaneous coronary intervention during hospital stay, diagnosis at discharge (ST-segment elevation MI/non-ST-segment elevation MI), coronary artery bypass grafting and body mass index.

P values for interactions between pain and sex and between pain and age were 0.041 and 0.230, respectively, for all-cause mortality. For MACE, p values for interactions between pain and sex and between pain and age were 0.201 and 0.334, respectively.

## 4 Discussion

### 4.1 Principal findings

Our study, based on 98,441 MI patients, is by far the largest to date and with the longest follow-up period to report associations between self-reported pain post-MI and MACE and all-cause mortality. We observed an increasing risk for these outcomes with the severity of self-reported pain. Moderate and extreme pain combined was more common among women (54 %) than men (40 %). However, for both outcomes, the relative risk associated with pain appeared higher in men compared to women (but sex interaction was only statistically significant for the all-cause mortality outcome). We noticed 24 % and 70 % adjusted average excess rates for all-cause mortality among men with moderate pain and extreme pain, respectively. Moreover, we could demonstrate that the relative risks were time dependent. The rates were most pronounced shortly after the 1-year visit post-MI and decreased slowly but the rate was still above 50 % adjusted excess rate of all-cause mortality even up to 14 years after the 1-year visit post-MI for men with extreme pain. Adjusted HRs for pain versus all-cause mortality and MACE in patients with lower cardiovascular risk defined, among other factors, by absence of chest pain, were comparable to the corresponding HRs in the main sample. The population attributable fraction associated with pain was similar to that of most established cardiovascular risk factors, such as smoking, diabetes, and hypertension, emphasizing that our findings may have important public health implications.

### 4.2 Comparison of the literature

In our previous study [26] with a shorter follow-up period (up to 8.5 years), smaller study sample and fewer events, adjusted hazard ratios (not stratified by sex) for all-cause mortality appeared somewhat higher compared to the present study (HR 1.35 (95 % CI 1.18–1.55) and 2.06 (95 % CI 1.63–2.60) for moderate pain and extreme pain, respectively). Since we demonstrated declining hazard ratios over time in this study, it is expected that average hazard ratios for a shorter follow-up period would be higher than those for a longer follow-up period.

In the study by Pocock et al.[35] with 8 978 MI patients, the association between pain (1–3 years post-MI) and all-cause mortality (at two-year follow-up) was as follows: rate ratio 1.12 (95 % CI 0.90–1.39). However, the authors found that the association between pain and a composite of cardiovascular death, MI, stroke, and unstable angina requiring urgent revascularization revealed a rate ratio of 1.35 (95 % CI 1.14–1.60). Our study size was ten times that of the study by Pocock et al.[35], our follow-up time was longer, and we used information on pain severity.

Population attributable risk (PAF) for pain and outcome all-cause mortality was 8.3 % and 6.3 % for men and women, respectively, which was comparable to an estimate (8.5 %) for chronic pain in a population-based study from UK Biobank in individuals without cardiovascular disease at baseline [15]. Thus, our findings support the notion that chronic pain is a cardiovascular risk factor with important public health implications throughout the cardiovascular continuum, in apparently healthy individuals with low risk as well as in high-risk individuals with prevalent cardiovascular disease.

### 4.3 Potential mechanism

At the baseline of the present study, we saw a clear pattern of a worsened cardiovascular risk profile with increasing levels of general pain. Despite this, the association between pain and MACE/all-cause mortality was still substantial and significant after adjustment for all these established cardiovascular risk factors, treatments, and MI type. This indicates that other unmeasured factors such as socioeconomic factors [[36], [37], [38]], sleep disturbances [39,40], chronic stress [22,23,41], co-morbidities such as rheumatoid arthritis [42] or low-grade inflammation [16,17,43] could mediate or confound the associations between pain and cardiovascular events. Also, there was no data on common pain medication, such as non-steroidal anti-inflammatory drugs and opioids, which have been shown to increase cardiovascular risk [44,45]. However, when using E-values to assess the impact of unmeasured confounding factors we found them unlikely to substantially affect the results. This observational study cannot draw unambiguous conclusions about causality but the dose–response relationship between pain severity and outcomes may reflect a causal link.

We observed stronger relative risks between pain and all-cause mortality in men than in women. This sex difference [46] was most pronounced for patients with extreme pain. Similar results are seen in the general population [47,48] but the underlying mechanism remains unclear.

### 4.4 Clinical implications

Our data suggest that clinicians treating post-MI patients should recognize general pain as a prognostic factor and take this into account when tailoring individualized cardiac rehabilitation and secondary prevention strategies, even for patients with lower cardiovascular risk (e.g. without chest pain) than the general MI population. Our PAF estimates indicate that 6 %-8 % of the total burden of all-cause mortality may be attributed to pain. These estimates were similar to PAF for the well-known risk factors current smoking, diabetes, and hypertension. This further highlights the public health significance of our findings and underscores the potential benefits of enhanced preventive treatment, such as integrated pain management programs, for individuals experiencing pain.

### 4.5 Strengths and limitations

The large and representative sample of MI cases over a 16-year period is a key strength of this study. This extensive sample allowed us to explore potential interactions between age and pain, as well as sex and pain, on outcomes. Another strength is that pain was assessed one year after MI, minimizing the influence of acute chest pain. Additionally, the long follow-up period, extending up to 16 years after the visit 1-year post-MI, enabled us to investigate temporal changes in the association between pain and all-cause mortality. The pain dimension of EQ-5D is ambiguous, as it combines two distinct aspects of health—pain and discomfort—even though it predominantly reflects pain [49]. One limitation is the fact that we did not have any information on pain localization, widespreadness and duration. However, among the 51,337 patients who reported no pain at the 2-month follow-up after MI, 73 % remained pain-free at the visit 1-year post-MI. Similarly, 65 % of those who experienced pain at the 2-month follow-up continued to report pain at the visit 1-year post-MI, indicating chronicity of pain. Among those with new pain 1-year post-MI, 80 % did not exhibit chest pain (based on CCS). This finding suggests that for the majority of patients experiencing new pain, heart-related issues were likely not the underlying cause.

However, since we were unable to explicitly measure chronic pain, the association between pain and outcomes may have been underestimated. When measuring pain after MI there is a possible interplay between general pain and chest pain. Importantly, our results were essentially unaltered when we restricted the sample to patients with lower cardiovascular risk, defined, among other factors, by the absence of chest pain. This argues against a higher burden of ischemic chest pain as an explanation of our findings.

Our study design excluded mortality within the first-year post-discharge, and only patients who attended a secondary prevention clinic 1-year after discharge and performed an EQ-5D measurement of pain were included. We were able to compare included and non-included patients who were alive 1-year post-MI, revealing lower subsequent mortality among those included. We noted a tendency to less pain troubles among the included than the non-included patients, albeit only based on 14 % of non-included patients and 90 % of the included patients with data on pain troubles at the two-month follow-up. It is likely that pain troubles were more frequent among those not attending any of the follow-ups. Given the higher subsequent mortality in the non-included patients versus the included patients, our hazard ratios were probably underestimated.

The upper age limit of 79 years restricts our findings from being applicable to older patients. Given that comorbidities affecting pain may be more common in older individuals, further research including more comprehensive comorbidity data would be necessary to explore this group. Our study sample being exclusively Swedish, can limit the generalizability of our findings. However, in Sweden, ethnicity is not recorded in official registers or epidemiological cohorts due to historical, legal, and ethical considerations. Therefore, we cannot present data on this issue.

The results may be partially influenced by reverse causation. To address this, we conducted sensitivity analyses excluding patients with less than 2 years of follow-up after the 1-year visit. The results remained largely unchanged, suggesting that the impact of reverse causation was minimal.

## 5 Conclusions

Pain one year after a MI is highly prevalent. Patients experiencing moderate to extreme pain one-year post-MI had higher rates of mortality and MACE over a 16-year follow-up compared to those without pain, with a stronger association in men than in women for outcome mortality, particularly for extreme pain. MI patients with lower cardiovascular risk compared to the general MI population might not receive adequate attention in healthcare. However, pain remains a significant risk factor for recurrent cardiovascular events and mortality for this group.

## Acknowledgement of grant support

Johan Ärnlöv was supported by Swedish research council (2023-06118).

## CRediT authorship contribution statement

**Lars Berglund:** Writing – review & editing, Writing – original draft, Visualization, Software, Project administration, Methodology, Investigation, Formal analysis, Data curation, Conceptualization. **Ann-Sofie Rönnegård:** Writing – review & editing, Writing – original draft, Visualization, Methodology. **Bertil Lindahl:** Writing – review & editing, Methodology, Investigation, Conceptualization. **Björn Äng.:** Writing – review & editing, Writing – original draft. **Torsten Gordh:** Writing – review & editing. **Kristina Hambraeus:** Writing – review & editing. **Johan Ärnlöv:** Writing – review & editing, Writing – original draft, Visualization, Project administration, Methodology, Funding acquisition, Conceptualization.

## Declaration of competing interest

The authors declare the following financial interests/personal relationships which may be considered as potential competing interests: Johan Ärnlöv has received lecturing fees from AstraZeneca and Boehringer Ingelheim and served on advisory boards for AstraZeneca, Boehringer Ingelheim and Astella, all unrelated to the present project. The authors do not report any additional disclosures in relation to this study.
